# Fulminant anti-α-amino-3-hydroxy-5-methyl-4-isoxazolepropionic acid receptor GluR1 antibodies encephalitis in a Chinese boy: a case report

**DOI:** 10.1186/s12887-022-03356-5

**Published:** 2022-05-17

**Authors:** Wei Han, Jiannan Ma, Li Jiang, Min Cheng

**Affiliations:** Department of Neurology Children’s Hospital of Chongqing Medical University, National Clinical Research Center for Child Health and Disorders, Ministry of Education Key Laboratory of Child Development and Disorders, China International Science and Technology Cooperation Base of Child Development and Critical Disorders, Chongqing Key Laboratory of Pediatrics, No.136 Zhongshan 2nd Road, Chongqing, 400014 China

**Keywords:** Autoimmune encephalitis, AMPAR encephalitis, GluR1 receptor, Rhabdomyolysis

## Abstract

**Background:**

Anti-α-amino-3-hydroxy-5-methyl-4-isoxazolepropionic acid receptor (AMPAR) encephalitis is a rare autoimmune synaptic encephalitis associated with autoantibodies that cause a selective decrease in surface expression and changes in receptor localization. Anti-AMPAR encephalitis is poorly recognized, especially in children, and its clinical phenotype is incompletely described.

**Case presentation:**

We report a case of anti-AMPAR GluR1 antibody-mediated autoimmune encephalitis in a 12-year-old male. The patient manifested as a fulminant course, with ataxia, cerebellar degeneration at the onset, and rapidly evolved into hyperthermia, coma and rhabdomyolysis. Antibodies against AMPAR GluR1 receptors were detected in the cerebrospinal fluid by cell-based assay. Diffuse slow waves were found by electroencephalograph, and the left cerebellar vermis and hemisphere were affected on brain magnetic resonance imaging (MRI). The patient was treated with intravenous immunoglobulin (IVIG), methylprednisolone combined with plasma exchange. Symptoms were alleviated after immunotherapy and the patient sustained clinical improvement. This is the first time that acute rhabdomyolysis symptom has been identified in a pediatric patient with anti-AMPAR encephalitis.

**Conclusions:**

This case expands the clinical spectrum of anti-AMPAR encephalitis and highlights that despite poor clinical manifestation at the outset, recovery remains possible.

**Supplementary Information:**

The online version contains supplementary material available at 10.1186/s12887-022-03356-5.

## Background

AMPAR is an ionotropic receptor belonging to the family of glutamate receptors that are located on the post-synaptic membrane and play essential roles in synaptic plasticity, memory, and learning [1]. AMPAR has four subunits—GluR1, GluR2, GluR3, and GluR4—and are mainly located in the cerebellum, cerebral cortex, and hippocampus.

Anti-AMPAR encephalitis is a rare, poorly recognized entity with varied clinical presentations including limbic encephalitis (LE), psychosis, and is frequently accompanied with neoplasms or paraneoplastic syndrome [2]. To date, only a few individual cases of pediatric anti-AMPAR encephalitis have been reported presenting with LE or seizure [3–5], with limited clinical experience. Fulminant encephalitis is a severe form of encephalitis, presenting a rapid neurological deterioration and evolving into loss of consciousness, hyperthermia, psychiatric and cognitive impairment [6]. Fulminant anti-AMPAR GluR1 antibody-mediated autoimmune encephalitis is very rare and most of the previously described patients had antibodies against GluR2. Herein, we aimed to report clinical features and follow-up for a patient with fulminant anti-AMPAR GluR1 antibodies encephalitis.

### Case Presentation

A 12-year-old Chinese male was admitted to Children’s Hospital of Chongqing Medical University with an 8-day history of vomiting and a 3-day history of dizziness, blurred vision, and gait disturbance. No fever, seizure, sleep disorders, speech or abnormal behavior was observed. Initial neurological examinations showed unstable standing, positive finger-to-nose and heel-to-knee-to-shin tests and stage 5 of bilateral limbs muscle power. There was no recent history of infections such as herpes simplex virus, Japanese encephalitis virus or SARS-CoV2. There was no any family history of similar condition or other types of autoimmune diseases. The patient did not take any medications prior to the development of symptoms and did not have any unusual environmental exposures.

**Fig. 1:**
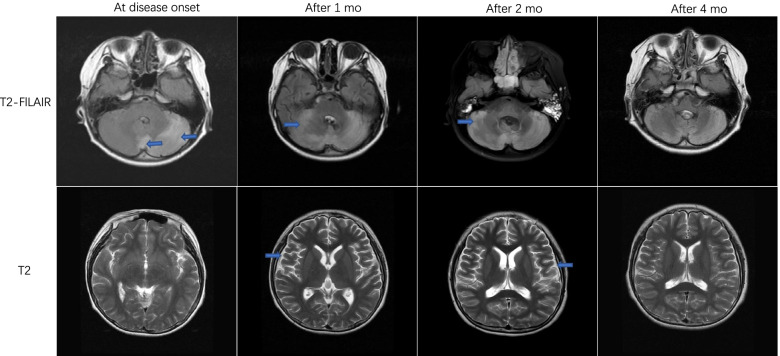
Representative brain magnetic resonance imaging at disease onset, after 1 month,2 months and 4 months in the fulminant encephalitis patient. The lesions of cerebellar hemisphere and cerebellar vermis(arrowhead) were identified in T2/fluid-attenuated inversion recovery (FLAIR) imaging. T2 Signal were shown cerebral parenchymal atrophy(arrowhead) after 1 month of disease and basically recovered after 4 months

Routine blood and urine tests (Table S1), genetic metabolism screening, C-reactive protein, and autoantibody tests, including anti-nuclear antibody and anti-dsDNA, were normal. The cerebrospinal fluid (CSF) cell count was 310 (WBC cells: 230;90% mononuclear and 10% multinuclear cells), whereas CSF total protein (0.64 g/L), glucose (3.18 mmol/L), and chloride (120 mmol/L) levels were normal. PCR for herpes simplex virus, coxsackievirus, measles virus, varicella zoster virus and enterovirus in the serum and CSF were all negative. Diffuse delta waves were found by electroencephalography in awake stage (Figure S1). An initial brain MRI revealed T2 Fluid-Attenuated Inversion Recovery (FLAIR) hyperintense signals within the left cerebellar vermis and hemisphere (Fig. 1 and Figure S2). The initial diagnosis was cerebellar encephalitis. Intravenous immunoglobulin was administered at 400 mg /kg/day for five days, and dexamethasone was administered at 0.3 mg/kg/day for six days.

Four days after admission, the patient developed delirium and urinary incontinence with aggravated dystonia and strong resting tremors on both limbs. Eight days after admission, the patient had a high-grade fever up to 39.2 °C and became unconscious. Repeat serological testing nine days after admission showed a creatine kinase (CK) of 15,153U/L, a creatine kinase myocardial band of 8.07 U/L, a myoglobin of > 1,000 μg/L, and an aspartate aminotransferase of 192 U/L, and urinalysis showed occult blood “ +  +  + ”; moreover, the CK value gradually increased to 98,076 U/L.

**Fig. 2:**
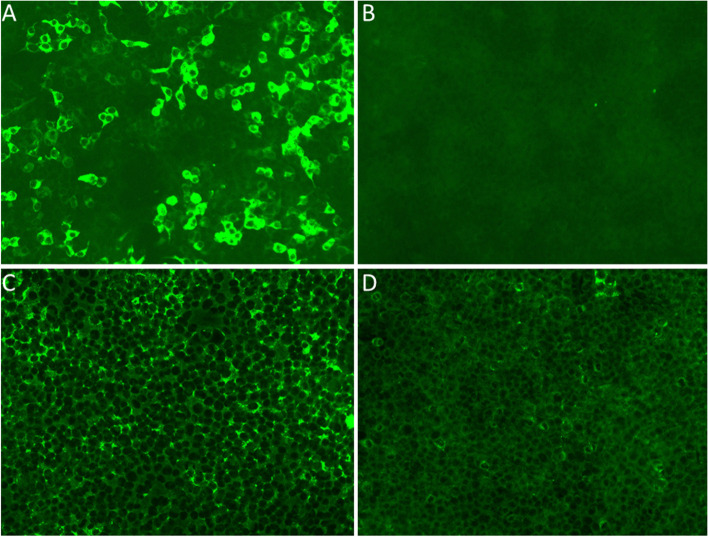
Neuroimmunological investigations the patient’s CSF (**A**) and serum (**C**) antibodies against the AMPAR GluR1. (**B**) and (**D**) were CSF and serum negative control respectively

Due to the new onset symptom of delirium and dystonia, the diagnosis of autoimmune encephalitis was suspected, antibodies against cell surface or synaptic proteins were assessed in the serum and CSF obtained using transfected HEK-293 cell by the cell-based assay and indirect immunofluorescence method (KingMed Diagnostics, Guangzhou, China). The results showed high levels of CSF (1:100) antibodies against the α-amino-3-hydroxy-5-methyl-4-isoxazolepropionic acid receptor (AMPAR) GluR1 but no antibodies in the serum (Fig. 2). Antibodies against AMPAR GluR2, N-methyl-D-aspartate receptor, GABA B type receptor, leucine-rich glioma inactivated protein 1 (LGI1) and Casper were negative both in serum and CSF (Figure S3). Paraneoplastic syndrome antibody (including GAD65, Zic, Tr (DNER), SOX1,Ma2,Ma1,Amphiphysin,CV2,Yo,Hu and Ri) test was negative in the serum. Tumor markers (including CEA, AFP, Ferritin and NSE), bone marrow puncture test, Chest and abdomen computed tomography (CT) were normal.

The CK value and urinalysis resolved following alkaline urine and plasma exchange, which was applied twice on the 12th and 14th days after admission. Intravenous methylprednisolone was initiated at 15 mg/kg/day for three days and then 8 mg/kg/day for three days, followed by oral prednisone at a dose of 1 mg/kg/day. The patient gradually recovered consciousness. A modified Rankin Scale (mRS) score of 4 and severe neurological deficits was observed, and the patient was transferred to the rehabilitation department 28 days after admission. Follow-up brain MRI FLAIR, one month after disease onset, showed that the cerebellar vermis lesions have partially absorbed but revealed new lesion in the right cerebellar hemisphere. Moreover, lesion evolved to cortical atrophy at 1 month but recovery at 4 months after onset (Fig. 2). Four-month later, mRS was 1, with ataxia and mild cognitive disorder.

## Discussion and Conclusion

Dizziness and ataxia at the onset of the adult patients with Anti-AMPAR encephalitis account for around 18% of previous reports [7], and coma, hyperthermia, rapid diffuse cortical atrophy also reported in 2 adult fulminant encephalitis cases before [6]. To our knowledge, this is the first time reported fulminant anti-AMPAR GluR1 encephalitis in children. Cerebellar signs in the case probably due to the strong expression of GluR1 receptor mainly located in the cerebellum.

Another noteworthy feature of this case is the presence of acute rhabdomyolysis after immunotherapy. Rhabdomyolysis is a critical syndrome in which destroyed muscle fibers degrade, and muscle components leak and are released into the circulation, resulting in acute renal failure. Acute rhabdomyolysis has been observed in other autoimmune encephalitis, particularly in anti-NMDAR encephalitis; however, to our knowledge, this is the first case in the literature where prominent rhabdomyolysis was observed in anti-AMPAR antibody encephalitis. A previous study indicated that rhabdomyolysis is a frequent complication in anti-NMDAR encephalitis [8]. Li et al. [9] have reported anti-voltage-gated potassium channel-complex antibody encephalitis complicated with rhabdomyolysis. However, the precise mechanisms underlying acute rhabdomyolysis induction remain unknown. It has been indicated that immunotherapy and the use of dopamine receptor blockers (DRBs) increase the risk of rhabdomyolysis [8]. In our case, we suspected it probably due to status dystonicus. Alternatively, it could be due to the aggravation of dyskinesia, high fever, and hypersensitivity to DRB. No obvious associations were observed between disease severity and rhabdomyolysis development or CK level as observed in our case. Acute rhabdomyolysis may be a rare complication of fulminant forms of anti-AMPAR encephalitis.

No evidence of neoplasm or spectrum of paraneoplastic syndrome has been found. However, long-term follow-up is still needed because neurological symptoms may develop prior to an oncologic diagnosis being made.

Due to the limited number of fulminant cases reported in the literature, the reasons why some patients develop severe encephalitis, and what causes such variable presentation, are not known. Our case report highlights the severity and complexity of anti-AMPAR encephalitis. It is important for pediatricians to recognize this rare disease because, despite severe clinical deficits of anti-AMPAR fulminant encephalitis, recovery is still possible with effective and timely treatment.

## Supplementary Information

**Additional file 1.** Supplementary Data 

## Data Availability

All data generated or analysed during this study are included in this published article and its supplementary information files.

## Abbreviations

AMPAR: Anti-α-amino-3-hydroxy-5-methyl-4-isoxazolepropionic acid receptor
LE: Limbic encephalitis
CSF: Cerebrospinal fluid
FLAIR: Fluid-Attenuated Inversion Recovery
CK: Creatine kinase
mRS: Modified Rankin Scale
DRBs: Dopamine receptor blockers
