# Supplementary material for: Fulminant anti-α-amino-3-hydroxy-5-methyl-4-isoxazolepropionic acid receptor GluR1 antibodies encephalitis in a Chinese boy: a case report
Source: BMC Pediatr. 2022 May 17;22:287. doi: 10.1186/s12887-022-03356-5 (PMC9110937; doi:10.1186/s12887-022-03356-5)
Supplement: Supplementary file 1 — Additional file 1. Supplementary Data [file 12887_2022_3356_MOESM1_ESM.docx]

**SUPPLEMENTARY DATA**


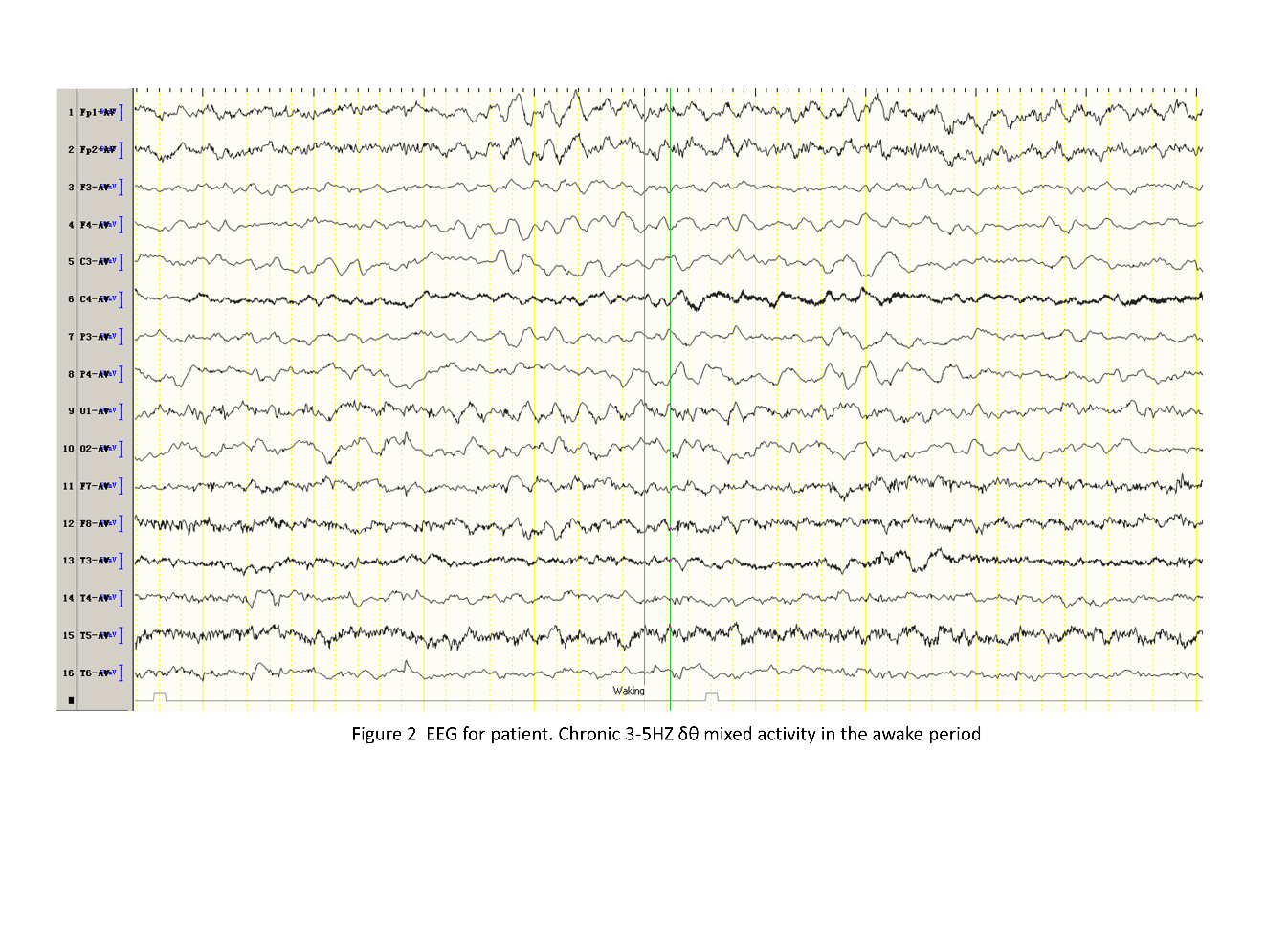


Figure S1. EEG for patient. The δθ wave mixed activity in the wake period was found in electroencephalographs


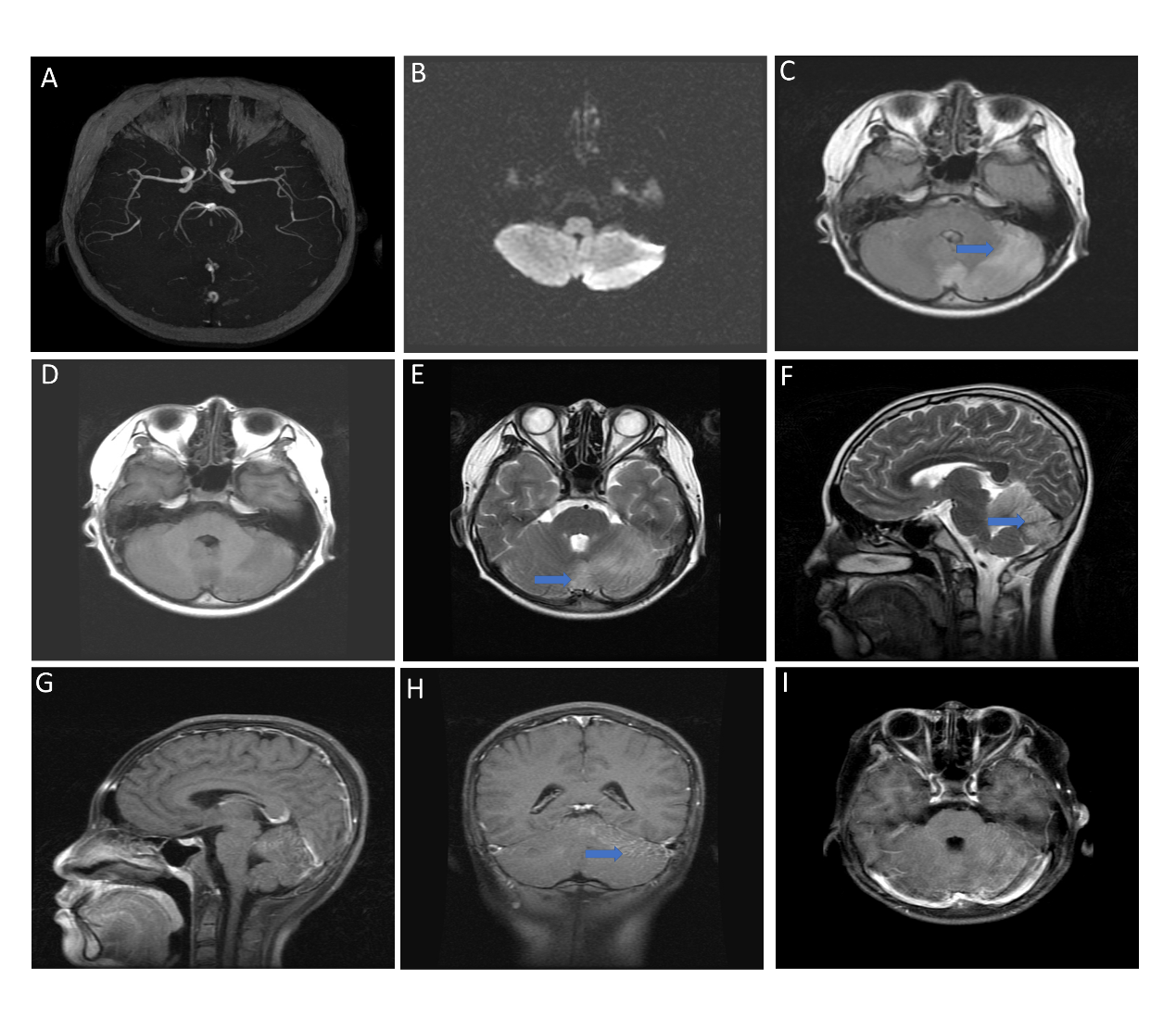


Figure S2. Brain MRI for the patient at onset of disease. MRA shows front and rear traffic arteries was unclear (A). DWI (B), T2FLAIR(C), T1WI(D), T2WI (E,F) and T1WI enhancement (G,H,I) identified left cerebellar vermis and left cerebellar hemisphere lesions (arrowhead).


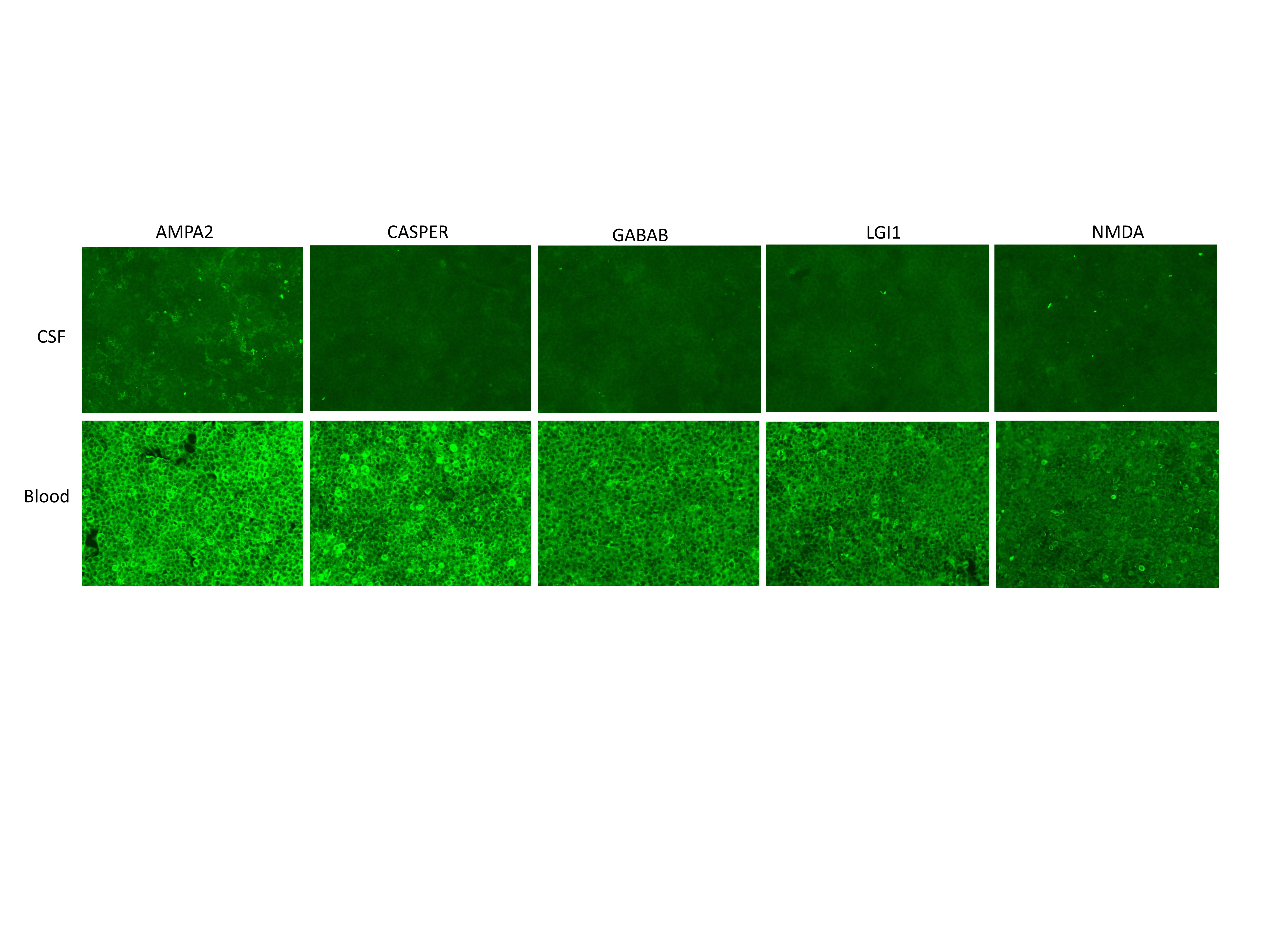


Figure S3 Neuroimmunological investigations CSF and serum antibodies against the AMPAR GluR2, CASPER,GABAB,LGI1 and NMDA respectively.

Table S1. Routine Blood tests and urinalysis of the patient

| Day after admission | WBC,10^9^/L | PLT,10^9^/L | Hb,g/dL | U-occult blood | U-RBC,cells/mL |
| --- | --- | --- | --- | --- | --- |
| 1 | 4.91 | 231 | 146 | - | - |
| 8 | 10.41 | 228 | 117 |  |  |
| 9 | - | - | - | +++ | 200 |
| 11 | - | - | - | +++ | 200 |
| 13 | 16.24 | 234 | 116 |  |  |
| 18 | - | - | - | - | - |
| 20 | 9.83 | 290 | 111 |  |  |
| 24 | 6.65 | 272 | 126 | - | - |
